# Processed human amniotic fluid retains its antibacterial activity

**DOI:** 10.1186/s12967-019-1812-8

**Published:** 2019-03-01

**Authors:** Yong Mao, Jan Pierce, Anya Singh-Varma, Michael Boyer, Joachim Kohn, Jo-Anna Reems

**Affiliations:** 1 New Jersey Center for Biomaterials, Rutgers University, 145 Bevier Rd., Piscataway, NJ 08854 USA; 2 Department Hematology & Hematologic Malignancies, University of Utah, Salt Lake City, USA; 3 Cell Therapy and Regenerative Medicine Facility, University of Utah, 676 Arapeen Drive, Suite 300, Salt Lake City, UT 84108 USA

**Keywords:** Amniotic fluid, Antibacterial, ESKAPE, Immunoprecipitation

## Abstract

**Background:**

Human amniotic fluid (AF) contains numerous nutrients, trophic factors and defense proteins that provide a nurturing and protective environment for fetal development. Based on reports that AF has antibacterial, anti-inflammatory and regenerative properties, we designed a novel method to process AF for use in clinical care.

**Methods:**

Six randomly selected lots of processed AF (pAF) were examined to determine whether they retained their antibacterial activity against a panel of wound-associated pathogens *E. faecium, S. aureus, K. pneumoniae, A. baumannii, P. aeruginosa*, and *E. aerogenes* (ESKAPE). To identify proteins in pAF that might be responsible for its antibacterial activity, three different lots of pAF were analyzed with quantitative cytokine arrays that consisted of 400 unique human proteins. One protein identified by microarrays, lactoferrin, and a second prominent antibacterial protein that was not identified by microarrays, lysozyme, were examined by depletion experiments to determine their contribution to the antibacterial activity of pAF.

**Results:**

All six lots of pAF exhibited antibacterial activity against ESKAPE microorganisms, especially against the pathogens predominately found in chronic wounds (i.e. *S. aureus* and *P. aeruginosa*). Thirty-one of the peptides on the microarray were annotated as having antibacterial activity and 26 of these were detected in pAF. Cystatin C and lactoferrin were among the most highly expressed antibacterial proteins in pAF. Cystatin C and lactoferrin were confirmed by ELISA to be present in pAF along with lysozyme. Immunoprecipitation of lactoferrin and lysozyme reduced, but did not abolish the antibacterial activities of pAF.

**Conclusion:**

Our data demonstrate that pAF maintains antibacterial activity via the preservation of antibacterial proteins against a broad spectrum of wound-associated pathogens.

## Background

The first use of human placental membranes for the treatment of diseases occurred with Chinese and Japanese traditional medicines centuries ago, and around the turn of the 20th century for Western cultures [1, 2]. Since these early applications, investigators identified that there are antibacterial and other paracrine factors in amniotic membrane that modulate the wound healing process [1, 3]. Like amniotic membrane, amniotic fluid (AF) plays important roles in the development and the protection of the fetus [4]. One property of AF that helps to defend the fetus against pathogens is its antibacterial activity [5–7]. AF contains numerous defense proteins and cytokines and antibacterial peptides, like cystatin C, lactoferrin and lysozyme [8–11]. Other components that are present in AF that confer antibacterial activity include transferrin, β-lysin, peroxidases, immunoglobulins, and zinc-peptide complexes [8, 12–14]. Also, chemokines such as CXCL1 and CXCL14 with known antibacterial activities are present in AF [15].

Amniotic fluid is a complex matrix that can be viewed as containing multiple fractions: (1) an insoluble fraction that consists of components like lanugo, vernix and cellular elements; (2) a soluble fraction that is made up of carbohydrates, proteins, lipids, electrolytes and metabolites and; (3) a fraction that contains extracellular vesicles (ECVs). Based on the knowledge that cytokines, growth factors and chemokines play major roles in activating endogenous mechanisms to facilitate repair and regeneration, we developed a novel approach to produce a processed AF (pAF) that eliminates a majority of its insoluble components (i.e. lanugo, vernix and cells) [16]. Interestingly, despite the removal of these insoluble components that include the stem cells, pAF supports the healing of chronic and acute wounds (unpublished observation). This observation suggests that active ingredients retained within the soluble fraction of pAF have properties capable of facilitating wound healing. From a previous study, we reported that 76% of a total of 400 proteins that we tested were present in pAF and that a majority of these proteins had roles in host defense [16]. Among the host defense peptides are proteins known for their role in the inflammatory response, innate immunity, immune modulation, and/or as having antibacterial activity.

Chronic wound environments are often colonized with microbes [17] and this colonization by microorganisms likely results in delayed healing due to infection [18, 19]. Delays in the healing process for chronic wounds is also attributable to prolonged inflammation caused by a lack of proper growth factors and cytokines [20]. Consequently, therapies that have antibacterial and anti-inflammatory activity would be a good choice for treating wounds. Recognizing that pAF facilitates the healing of chronic and acute wounds, the goals of this study were to assess the antibacterial activity of unprocessed AF relative to pAF, to evaluate the antibacterial activity of pAF from different donors, and to begin to characterize the role of specific antibacterial proteins in pAF against a panel of wound-associated pathogens.

## Materials and methods

### Collection and processing of amniotic fluid

Donor consent, screening and infectious disease testing of amniotic fluid (AF) were previously described [16]. The collection and processing of AF were also previously described [16]. Prior to processing AF, several aliquots were removed and stored frozen at − 80 °C (i.e. pre-processed AF). Upon the completion of the final processing step, aliquots of pAF were removed and the samples were maintained at − 80 °C until the time of use.

### Bacterial culture and preparation of inoculums

Clinical isolates of ESKAPE bacterial strains were purchased from ATCC (Manassas, VA): *E. faecium* ATCC^®^ 51559™, *S. aureus* ATCC^®^ 25923™, *K. pneumoniae* ATCC^®^ 700603, *A. baumannii* ATCC^®^ 49466™, *P. aeruginosa* ATCC^®^ 15692™, and *E. aerogenes* ATCC^®^ 49469™. All bacterial strains were cultured and maintained as instructed by ATCC^®^. Optimal culture media used are tryptic soy broth (TSB) for *S. aureus*, *P. aeruginosa, E. aerogenes*, *A. baumannii*, brain heart infusion (BHI) medium for *E. faecium* and nutrient broth for *K. pneumoniae*. Preparation of bacterial inoculum was performed as described previously [21]. Briefly, bacteria were cultured in their defined optimal culture medium at 37 °C with shaking until optical density reached 0.2 to 0.6 at 600 nm (OD_600_). The number of colony forming units (CFUs) for each strain was estimated based on an OD_600_ = 1.0, which corresponds to 10^9^ CFU/mL. To prepare the inoculum for antibacterial assays, the bacterial stocks were serially diluted with culture medium or pAF to approximately 1 × 10^3^ CFU/mL of bacteria. For each experiment, the actual CFU of each inoculum was determined by preparing serial dilutions and then plating onto TSB agar plates (BD, Franklin Lakes, NJ).

### Inhibition of bacterial growth by pAF

An inoculum of *P. aeruginosa* or *S. aureus* (1 × 10^3^ CFU/mL) was added to pAF that had been prepared by diluting pAF in TSB. The cultures were incubated at 37 °C for 24 h. The growth of bacteria was monitored using an alamarBlue assay (ThermoFisher Scientifics; Waltham, MA) by following the manufacture’s protocol. The fluorescent intensity was measured using a TECAN Spark 10 M plate reader (TECAN, Morrisville NC) at Ex560 nm/Em590 nm. The growth of bacteria in the presence of pAF was normalized to the growth in the absence of pAF.

### Quantification of bacterial growth in the presence of pAF

Bacterial growth was assessed as described with modifications [22]. Each strain of bacteria (1 × 10^3^ CFU/mL) was added to 1 mL of AF or 1 mL optimal culture medium. The cultures were incubated at 37 °C with shaking for 24 h. Serial dilutions were then prepared for each culture and plated onto TSB agar plates. CFUs were counted after overnight incubation at 37 °C. The antibacterial activity of pAF was expressed in log reductions, which was calculated as the log_10_CFU (Control) − log_10_CFU (pAF). Data from two independent experiments (n = 3 for each experiment) were pooled together to calculate the mean and standard deviation.

### Protein array

Quantitative Protein arrays were performed as previously described [16]. Briefly, pAF from three maternal collections were sent to RayBiotech to simultaneously and quantitatively measure the concentration of 400 human cytokines using the Quantibody ^®^ Human Cytokine Antibody Array 9000 (RayBiotech, In., Norcross, GA). Controls and serial dilutions of cytokine standards were prepared according to the manufacturer’s instructions and were added to chip wells. After processing the chips according to the manufacturer’s instructions, the chips were analyzed using the Quantibody^®^ Q-Analyzer software (RayBiotech, Inc.). Proteins were classified according to their biological function by surveying the Human Protein Reference Database (http://www.hprd.org/index_html), Cytokines & Cells Online Pathfinder Encyclopedia (COPE) http://www.copewithcytokines.de/), GeneCards^®^ (http://www.genecards.org/), and the biomedical literature in PubMed (http://www.ncbi.nlm.nih.gov/pubmed).

### Detection of human lysozyme, cystatin C and lactoferrin in pAF using ELISA

The presence of lysozyme, cystatin C and lactoferrin from different lots of pAF was quantified using quantitative sandwich ELISA assays according to the manufacturer’s instructions (Abcam, Cambridge, MA): Lysozyme (Human Lysozyme ELISA kit ab108880), Lactoferrin (Human Lysozyme ELISA kit ab200015), and cystatin C (Human Lysozyme ELISA kit ab179883).

### Immunoprecipitation (IP)

Selective depletion of lysozyme and lactoferrin from AF was accomplished by immunoprecipitation (IP) as described [22]. Briefly, rabbit polyclonal anti-lysozyme antibody (ab2408) and anti-lactoferrin antibody (ab15811) were purchased from Abcam. Anti-lysozyme antibodies (15 μg/mL) and anti-lactoferrin antibodies (4 μg/mL) were added individually (IP) or together (Co-IP) to 1 mL of AF. As a control, the same volume of PBS was added to 1 mL of AF. The samples were mixed at 4 °C for overnight. A 50 µL slurry of Protein A/G agarose plus resin: sc-2003 (Santa Cruz Biotechnology; Dallas, TX) was washed twice with 1 mL of PBS and mixed with each pAF with or without antibodies for 4 h at 4 °C. Mixtures were then centrifuged at 2000 rpm for 3 min and resulting supernatants were transferred to individual bacterial culture tubes. Inoculums of 1 × 10^3^ CFU of *P. aeruginosa* or *S. aureus* were added to each culture tube and incubated with shaking at 37 °C. After 24 h, CFUs for each culture were quantified by serial dilution plating as previously described [22].

### Statistical analysis

Each independent experiment contained 3 or more biological repeat samples (n ≥ 3), and data is presented as the mean ± standard deviation. One-way ANOVA with a Tukey’s multiple comparisons test was performed to determine statistical significance. Differences were considered significant at a p value of < 0.05.

## Results

### Processed AF inhibited the growth of *P. aeruginosa* and *S. aureus*

**Fig. 1:**
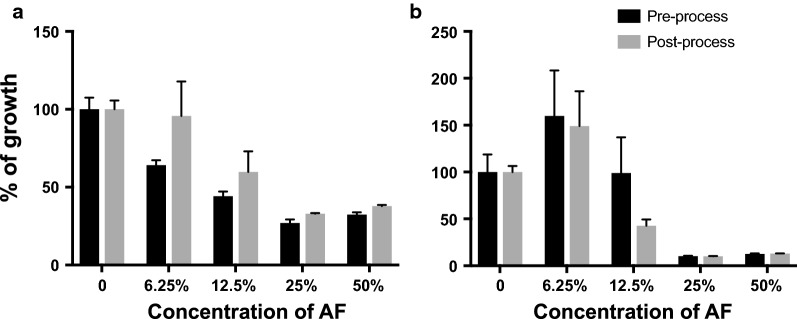
Relative antibacterial activity of AF before and after processing. The growth of *P. aeruginosa* (**a**) or *S. aureus* (**b**) in tryptic soy broth without AF or in TSB with different concentrations of AF were quantified using alamarBlue assay. The percent of growth reduction with AF/pAF is calculated as: fluorescent intensity of culture with AF/fluorescent intensity of control (no AF) × 100. Data are presented as mean ± SD (n = 3)

The antibacterial activities of unprocessed and processed AF were determined by measuring the growth of *P. aeruginosa* or *S. aureus* in the presence of either pre-processed AF or post-processed AF (Fig. 1). With increasing concentrations of unprocessed AF and pAF, the growth of either *P. aeruginosa* (Fig. 1a) or *S. aureus* (Fig. 1b) decreased 70% or 90%, respectively.

**Fig. 2:**
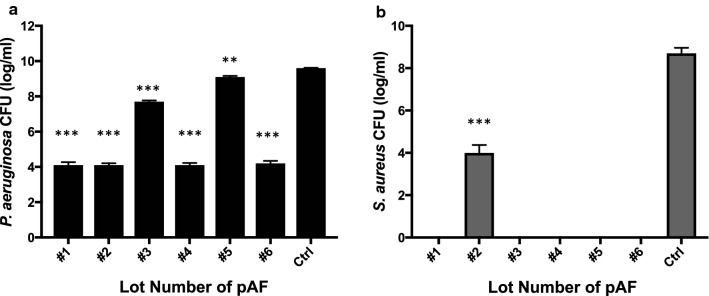
Antibacterial activity of processed AF. The growth of *P. aeruginosa* (**a**) or *S. aureus* (**b**) in different lots of pAF, PBS or were quantified by serial dilution and CFU counting after 24 h culture. Data are presented as mean ± SD for each lot (n = 3). Statistical analysis was performed to compare each lot of pAF with Control (Ctrl). **p < 0.01 and ***p < 0.005

Next, we examined the antibacterial activity of pAF from six different maternal collections against *P. aeruginosa* (Fig. 2a) and *S. aureus* (Fig. 2b). As was evident by a reduction in bacterial growth for both microorganisms, all six lots of pAF showed an antibacterial effect against *P. aeruginosa* (Fig. 2a) and *S. aureus* (Fig. 2b). Distinct lots of pAF appeared to have differential activity against different bacteria. Four out of six lots of pAF showed a growth inhibition of *P. aeruginosa* over 5 logs and two lots showed inhibition of less than 2 logs. On the other hand, five out of six lots showed a bactericidal effect against *S. aureus* (Fig. 2b).

### Processed AF has antibacterial activity against ESKAPE pathogens

After establishing that pAF retained the antibacterial activity of unprocessed AF, we examined the activity of pAF against a panel of Gram-positive and Gram-negative microorganisms that are known for their association with wound microenvironments and their tendency to have an antibiotic resistance nature. This panel of microbes includes: *E. faecium, S. aureus, K. pneumoniae, A. baumannii, P. aeruginosa*, and *E. aerogenes* and is often referred as the ESKAPE panel [23]. We quantified the growth of ESKAPE microbes in the presence of multiple lots of pAF (Table 1). The growth of each strain of bacteria in the presence of pAF was compared with the growth in optimal culture medium. Besides the inhibitory effects of pAF on the growth of *S. aureus* and *P. aeruginosa* as described in Fig. 2, all six randomly selected lots of pAF (lots 1–6) showed antibacterial activity against *E. aerogenes*. Among them, five lots of pAF showed strong activity against *E. aerogenes* (over 7 logs of growth reduction) and one lot (i.e. lot 5) had a lower activity against *E. aerogenes*. Four out of six lots reduced the growth of *K. pneumoniae* for over 4 logs while lots 3 and 5 of pAF showed weaker inhibitory activity against *K. pneumonia*. *A. baumannii* showed over 3 logs of growth reduction in the presence of all six lots of pAF except for lot 5, which had a weaker activity. The activities against *E. faecium* were comparable among different lots of pAF except for lot 3, which showed bactericidal effects against *E. faecium*. These results indicate that pAF has antibacterial activity and the antibacterial activity does vary by lot and by organism.

**Table 1:** Antibacterial activity of processed AF against ESKAPE pathogens

Lot #	*E. aerogenes*	*S. aureus*	*K. pneumoniae*	*A. baumannii*	*P. aeruginosa*	*E. faecium*
1	7.2 ± 0.1	8.7 ± 0.0	5.5 ± 0.1	3.3 ± 0.1	5.5 ± 0.2	2.3 ± 0.2
2	6.8 ± 0.0	4.7 ± 0.4	4.8 ± 0.3	5.2 ± 0.1	5.5 ± 0.1	2.6 ± 0.1
3	7.7 ± 0.6	8.7 ± 0.0	0.9 ± 0.0	4.8 ± 0.1	2.0 ± 0.1	9.2 ± 0.0
4	7.5 ± 0.1	8.7 ± 0.0	4.8 ± 0.7	3.7 ± 0.1	5.5 ± 0.1	2.4 ± 0.1
5	0.7 ± 0.1	8.7 ± 0.0	0.6 ± 0.1	0.8 ± 0.0	0.5 ± 0.1	1.8 ± 0.1
6	7.6 ± 0.2	8.7 ± 0.0	4.4 ± 0.4	5.0 ± 0.1	5.4 ± 0.1	3.0 ± 0.2

Expressed as a log reduction in growth against each bacterium; Log reduction = Log_10_ CFU_(Ctrl)_ − Log_10_ CFU_(AF)_

### Antibacterial proteins/peptides in AF

**Fig. 3:**
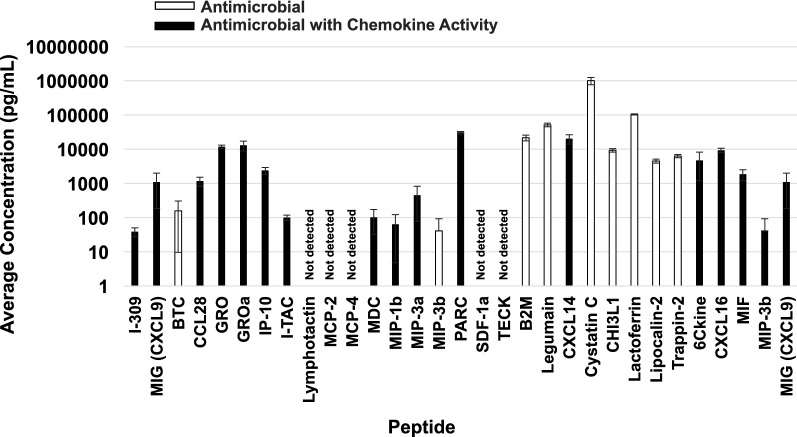
Proteins tested in pAF with known antibacterial activity. Quantibody^®^ Human Cytokine Antibody Arrays 9000 were used to examine the presence or absence of 400 different peptides in pAF. Of the 400 peptides that were tested (i.e. three lots other than six lots examined for antibacterial activity), an average of 304 ± 20 proteins (n = 3) were positively identified in pAF. Thirty-one of the 400 proteins that were tested were known as antibacterial peptides. Of the 31 antibacterial proteins that were tested, 26 were present in pAF. Among the detectable antibacterial peptides, 17 of them are also classified as chemokines (black bars) and 9 of them are not chemokines (white bars)

To evaluate potential proteins with antibacterial properties in pAF, we focused on antibacterial proteins that we identified from comprehensive protein array data sets that we previously published [16] (Fig. 3). The proteins identified were from 4 replicates of pAF from 3 different lots that were not used for the antibacterial testing of this study. Of 400 proteins tested, an average of 304 ± 20 (average ± SD) peptides were detected in the three lots of pAF. Within the list of 400 proteins tested, 31 peptides were annotated as possessing antibacterial properties. Of these 31 proteins tested, 26 or 84% of the proteins tested were present in pAF and 5 were not detectable. The results of the array showed that cystatin C (511 ± 122 ng/mL) and lactoferrin (52.7 ± 1.9 ng/mL) stood out as having the highest quantitative levels among antibacterial proteins (n = 3, mean ± SD). Also, of the 26 detectable antibacterial peptides, 17 of them were classified as chemokines (Fig. 3).

**Fig. 4:**
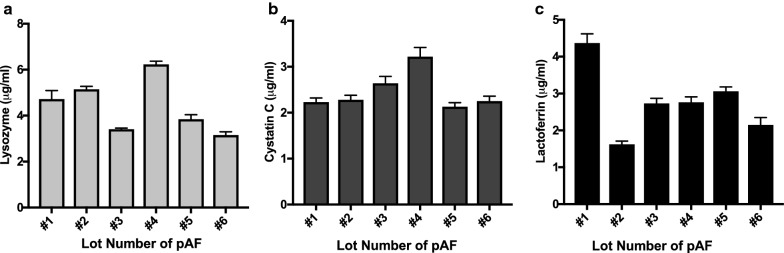
The presence of antibacterial proteins in multiple lots of AF. The levels of lysozyme (**a**), cystatin C (**b**) or lactoferrin (**c**) in different lots of AF were quantified using ELISA as described in Materials and methods. Data are presented as mean ± SD (n = 4)

In the next set of experiments, we validated the results of the array data by quantifying the levels of cystatin C and lactoferrin in pAF by using ELISA. We also selected to quantitate lysozyme levels in pAF as it had been previously reported by others to be present at high levels in AF and to confer antibacterial activity in AF [24]. The levels of lysozyme, cystatin C and lactoferrin in six different lots of pAF were quantified using ELISA (Fig. 4). Among the different lots of pAF, the levels of lysozyme, cystatin C, and lactoferrin varied. Three (i.e. lots 1, 2 and 4) of the four lots (i.e. lots 1, 2, 4, and 6) of pAF that had stronger antibacterial activity against *P. aeruginosa* (Fig. 2) and *K. pneumonia* also showed higher levels of lysozyme (Fig. 4a). This observation indicates that *P. aeruginosa* and *K. pneumoniae* may be sensitive to lysozyme in pAF. No apparent correlation between lysozyme levels (Fig. 4a) and antibacterial activity were noted against *S. aureus* (Fig. 2b). Levels of cystatin C were relatively comparable among the different lots of pAF with the exception of lot 4, which showed the highest level of cystatin C (Fig. 4b). No apparent correlation between cystatin C levels (Fig. 4b) and antibacterial activity were noted against *S. aureus* or *P. aeruginosa* (Fig. 2a, b). The levels of lactoferrin in different lots of pAF (Fig. 4c) showed a strong correlation with antibacterial activity against *S. aureus* among all six lots (Fig. 2b), where lot 2 with the lowest lactoferrin showed the lowest activity against *S. aureu*s. No specific correlation was identified between the level of specific antibacterial protein and activity against *E. aerogenes*, *A. baumannii* or *E. faecium*. Nevertheless, we observed that the highest levels of antibacterial proteins (i.e. lots 1 and 4) correlated with highest overall antibacterial activities.

### Antibacterial proteins contribute to the antibacterial activity of pAF

**Fig. 5:**
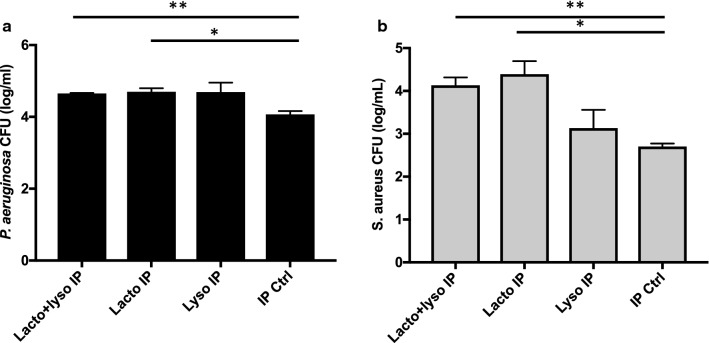
Immunoprecipitation (IP) of lysozyme and lactoferrin reduced the antibacterial activity of pAF. The growth of *P. aeruginosa* (**a**) or *S. aureus* (**b**) in pAF after IP or Co-IP of lysozyme and lactoferrin or after a mock IP was quantified. Data are presented as mean ± SD (n = 3), *p < 0.05, **p < 0.01

To evaluate the contribution of lactoferrin and lysozyme to the antibacterial activity of pAF, we selectively reduced the levels of these proteins in pAF by using immunoprecipitation (IP). Lot 2 was immunoprecipitated individually or in combination with antibodies against lactoferrin and with antibodies against lysozyme. As shown in Fig. 5 after IP with lactoferrin or lysozyme, the anti-*P. aeruginosa* activity of pAF was reduced by 4.9- and 4.6-fold, respectively (Fig. 5a). However, no further reduction of anti-*P. aeruginosa* activity was observed after a Co-IP with lactoferrin and lysozyme. On the other hand, while the depletion of lysozyme did not reduce anti-*S. aureus* activity of pAF, IP with lactoferrin or Co-IP with lactoferrin and lysozyme reduced the anti-*S. aureus* activity of pAF by over 28-fold (Fig. 5b).

## Discussion

Reports of stem cells in amniotic fluid (AF) has attracted the efforts of a number of groups for developing commercial products of AF for the treatment of wounds and other therapeutic applications [25, 26]. Based on our observation that there is a paucity of stem cells in AF and that expansion of the stem cells would be required to obtain therapeutic doses. We developed a processing method that removes a majority of the insoluble components (i.e. cells, lanugo and vernix) while retaining soluble components (i.e. cytokines, growth factors, chemokines, antibacterial peptides) and extracellular vesicles [16]. Our formulation of pAF is based on the hypothesis that the soluble components and extracellular vesicles found in AF will activate endogenous mechanisms to facilitate immune responses, tissue repair and regenerative events.

Recognizing that delayed healing can occur when wounds are colonized with microbes and that a therapy with antibacterial activity is advantageous towards healing infected wounds, this study determined whether AF depleted of its insoluble components retained its antibacterial activity. We found that pAF maintains its antibacterial activity relative to unprocessed AF and that the soluble components in pAF are key contributors to its antibacterial activity. This study also shows that differences in the antibacterial activity of different lots of pAF exist against different pathogens (Table 1). Given that a standardized method is used to process AF, it is more likely that the differences in the antibacterial activity of different lots is due to inherent differences among donors rather than to the manufacturing strategy.

Of the 31 known anti-microbial proteins that we tested, 26 of them are present in pAF. Cystatin C and lactoferrin were amongst the highest expressed antibacterial proteins that we tested. Cystatin C not only had the highest quantitative levels among antibacterial proteins, but was also among the top 5 most highly expressed proteins of ~ 300 proteins that we identified and quantified in pAF [16]. Cystatin C is a cysteine protease inhibitor [27] that is present in almost all tissue and body fluids and is reported to be a potent regulator of the inflammatory response. Cystatin C plays important roles in innate immunity by binding to components of the classical complement pathway and by modulating the actions of neutrophils via superoxide inhibition and chemotaxis [28–30]. Lactoferrin, is the most abundant protein in the whey fraction of human milk [31]. Lactoferrin plays a critical role in protecting the newborn infant from infection via its iron binding function that inhibits bacteria, fungus, viral and parasitic infections and by its anti-inflammatory and immunomodulatory activities [32–34].

Seventeen of the antibacterial proteins that we identified in pAF also possess chemokine activity, a property that involves directing the immune response to sites of injury and infection. For example, PARC, the most highly expressed chemokine in pAF triggers lymphocyte responses, but not neutrophils [35]. While GRO and GROa attract neutrophils [36]. Others of the identified chemokines present in pAF are proteins that can attract monocytes, eosinophils, and even basophils. Moreover, some chemokines have also been shown to have antibacterial activity that directly interfere with infectious agents [15]. The significance of this finding is that different chemokines in pAF may recruit different types of white cells to sites of infection, inflammation and/or injury to promote repair of the site.

To determine whether a relationship could be established between the levels of antibacterial proteins and their activity against specific pathogens, we focused our effort on 3 proteins found in AF, lactoferrin, cystatin C and lysozyme. Knowing that lactoferrin has antibacterial activity against *S. aureus* [37] and lysozyme has lytic activity against *P. aeruginosa* over *S. aureus* [24, 38], we were particularly interested in whether the levels of these proteins correlated with the bactericidal activity of pAF. We found that of six pAF lots tested, all lots except for lot 2 showed bactericidal activity against *S. aureus* (Fig. 2b) and lot 2 also had the lowest levels of lactoferrin (Fig. 4c). Likewise, lower levels of lysozyme in lots 3 and 5 (Fig. 4a) corresponded with lower anti-*P. aeruginosa* activity (Table 1). However, lower levels of lysozyme activity for lot 6 did not correspond to lower anti-*P. aeruginosa* activity. Also, the levels of cystatin C, lysozyme and lactoferrin in lot 6 were not higher than that of lot 5, but lot 6 had better overall antibacterial activity than lot 5. These results suggest that lactoferrin and lysozyme levels may contribute to differential antibacterial activities of pAF. However, we did not identify one single protein of the 3 antibacterial proteins we tested whose level in AF determines the antibacterial activity of AF. This suggests that other proteins/peptides with antibacterial proteins in pAF are likely to contribute to pAF’s antibacterial activity. Evidence that other antibacterial proteins in pAF are involved is supported by the results of our immunoprecipitation study. By specifically depleting lactoferrin and lysozyme from pAF, we showed that there was a direct involvement of lysozyme and lactoferrin in the antibacterial activity of pAF. However, the depletion of these proteins without abolishing the antibacterial activity of pAF indicates that additional antibacterial factors are involved in the antibacterial activity of pAF.

## Conclusion

The results of this study indicate that even after the removal of the insoluble components of human AF that the antibacterial activity of pAF is preserved and that a majority of the antibacterial activity of AF is contained within the soluble fraction. As we move towards controlled clinical studies to investigate the efficacy of using pAF in patients with burns and wounds, the antibacterial proteins identified under this study and their expression levels will be valuable in helping to assess their contributions in clinical outcome. In conclusion, pAF is rich in host defense proteins in which some are known for their antibacterial, antifungal, antiviral, anti-parasitic, anti-inflammatory and immunomodulatory activities.
